# Efficiency of two larval diets for mass-rearing of the mosquito *Aedes aegypti*

**DOI:** 10.1371/journal.pone.0187420

**Published:** 2017-11-02

**Authors:** J. G. Bond, A. Ramírez-Osorio, C. F. Marina, I. Fernández-Salas, P. Liedo, A. Dor, T. Williams

**Affiliations:** 1 Centro Regional de Investigación en Salud Pública (CRISP-INSP), Tapachula, Chiapas, Mexico; 2 El Colegio de la Frontera Sur (ECOSUR), Tapachula, Chiapas, Mexico; 3 Instituto de Ecología AC (INECOL), Xalapa, Veracruz, Mexico; Centro de Pesquisas René Rachou, BRAZIL

## Abstract

*Aedes aegypti* is a major vector of arboviruses that may be controlled on an area-wide basis using the sterile insect technique (SIT). Larval diet is a major factor in mass-rearing for SIT programs. We compared dietary effects on immature development and adult fitness-related characteristics for an International Atomic Energy Agency (IAEA) diet, developed for rearing *Ae*. *albopictus*, and a standardized laboratory rodent diet (LRD), under a 14:10 h (light:dark) photoperiod ("light" treatment) or continuous darkness during larval rearing. Larval development was generally fastest in the IAEA diet, likely reflecting the high protein and lipid content of this diet. The proportion of larvae that survived to pupation or to adult emergence did not differ significantly between diets or light treatments. Insects from the LRD-dark treatment produced the highest proportion of male pupae (93% at 24 h after the beginning of pupation) whereas adult sex ratio from the IAEA diet tended to be more male-biased than that of the LRD diet. Adult longevity did not differ significantly with larval diet or light conditions, irrespective of sex. In other aspects the LRD diet generally performed best. Adult males from the LRD diet were significantly larger than those from the IAEA diet, irrespective of light treatment. Females from the LRD diet had ~25% higher fecundity and ~8% higher egg fertility compared to those from the IAEA diet. Adult flight ability did not differ between larval diets, and males had a similar number of copulations with wild females, irrespective of larval diet. The LRD diet had lower protein and fat content but a higher carbohydrate and energetic content than the IAEA diet. We conclude that the LRD diet is a low-cost standardized diet that is likely to be suitable for mass-rearing of *Ae*. *aegypti* for area-wide SIT-based vector control.

## Introduction

*Aedes* (*Stegomyia*) *aegypti* (Linnaeus, 1762) was introduced into the New World from Africa, from where it subsequently spread globally to tropical and sub-tropical regions of the world [1, 2]. *Ae*. *aegypti* is the primary vector of arboviruses such as dengue (DENV), Chikungunya (CHIKV) and Zika (ZIKV) in the Americas [3, 4]. The prevention or reduction of the transmission cycles of these viruses is almost completely dependent on the control of mosquito vectors to restrict the frequency of contact between mosquitoes and humans [5]. Habitat elimination is the main approach to reduce mosquito populations, and chemical insecticides represent a second line of control against such vectors. However, frequent exposure to insecticides is related to adverse effects on populations of non-target organisms, as well as the development of resistance to insecticides in the vector populations [6]. Therefore, effective control of vector-borne diseases continues to represent a major challenge, requiring novel and innovative approaches.

One alternative for mosquito control is the sterile insect technique, SIT [7]. SIT is a species-specific method of insect control that relies on mass rearing, sterilization and release of large numbers of sterile males which, due to their high prevalence in the population, are the males that most frequently mate with wild females, induce sterility and thereby reduce the reproductive output of wild females. This leads to a decline in the vector population as long as the mass release of sterile males is maintained [8, 9].

The implementation of SIT requires the production of large numbers of sexually competitive males, their irradiation and release in an area-wide integrated management program [10]. Mass production of mosquitoes requires a suitable and inexpensive diet that provides adequate nutrients for proper larval development and the production of high-quality sterile males [11].

The purpose of this study was to compare two artificial diets for their effects on larval development and adult sexual competiveness in a laboratory setting. The International Atomic Energy Agency (IAEA) diet was previously recommended for larval development of *Aedes albopictus* [12]. The second diet was a laboratory rodent diet (LRD) used to fed laboratory mice in the Centro Regional de Investigación en Salud Pública (part of Mexico's National Institute for Public Health). These diets were evaluated to assess their suitability for mass rearing of *Aedes aegypti*.

In addition, larvae have a negative phototactic response [13] and are sensitive to stimuli that trigger defensive immersion, such as changes in light intensity and vibration [14, 15]. Therefore, we decided to evaluate larval rearing in darkness, which might increase larval feeding behavior and reduce the frequency of alarm responses and their associated energetic costs. For this, the effect of larval rearing on larval development and a selection of adult traits was compared under two light regimes; a standard photoperiod and continuous darkness achieved by covering trays with an opaque lid.

## Materials and methods

### Mosquitoes

The *Ae*. *aegypti* strain used in the experiments was collected as eggs in twelve localities along the Pacific coast of Chiapas state, Mexico. The *Aedes aegypti* colony was maintained for two generations under controlled conditions at 26 ± 2°C, 80 ± 5% relative humidity (RH), and photoperiod of 14:10 h (light: darkness). The methodology used to evaluate larval development and adult traits in the present study was the same as that described previously [12, 16], as outlined in the following sections. All experimental procedures were performed at 28 ± 1°C and 80 ± 5% RH.

### Diets

Two diets were compared. The first was a certified Laboratory Rodent Diet (LRD) LabDiet 5001 (PMI Nutrition International LCC, St. Louis, MO). The second was a diet developed by the International Atomic Energy Authority (IAEA 2). The composition of the two diets was as follows: LRD comprised maize, soybean meal, beet pulp, fish meal, oats, brewer's yeast, cane molasses, alfalfa meal, whey, wheat germ, porcine meat meal, wheat, salt, and a mixture of vitamins and minerals (manufacturer reported composition of the diet was 22% (wt/wt) crude protein; 4.5% crude fat; 6% crude fiber; 8% ash; 2.5% added minerals). The IAEA diet consisted of 25% (wt/wt) bovine liver powder, 50% tuna meal, and 12.5% brewer's yeast [12].

To compare the nutritional profiles of these diets, bromatological analyses were performed using standard techniques [17]. The following parameters were determined: a) ash (constant weight in stove 100°C for 4 hours followed by incineration at 550°C for 6 h); b) crude protein (digestion for 100 min at 380°C, distillation with 1% boric acid); c) fat (ether extraction for 10 h); crude fiber (acid/alkaline digestion and filtration through a Gooch crucible); d) total carbohydrate content (calculated by difference, taking into account protein, fat, water and ash); and e) energetic value [17]. For both diets, 4% liquid suspensions (wt/vol) were prepared by mixing the solid components in deionized water prior to use in feeding larvae.

### Effects of diets on larval development

To measure the effect of diets on *Ae*. *aegypti* larval development, groups of 750 first-instar larvae were counted and placed in plastic trays (38 x 25 x 6 cm) containing 500 ml of deionized water to obtain a density of 1.5 larvae per ml that was well within the range of densities used for optimized mass-rearing of *Ae*. *aegypti* (0.6–2.8 larvae/ml) [18] and *Ae*. *albopictus* (1.5–2.0 larvae/ml) [12, 19]. A 10 ml volume of liquid diet was added to each tray (equivalent to 0.53 mg diet per larva) each day, except for day 5 when double this amount was added to provide the required amount of food prior to pupation.

The effect of darkness on larval development was also determined. For this, half of the trays were covered with an opaque black plastic cover to exclude light or were left uncovered and exposed to light during the 14 h daily light cycle. Therefore, the experiment involved four treatment combinations: two diets, and a standard photoperiod (14h: 10h light:dark; hereafter named the "light" treatment), or continuous dark conditions during larval development. Three replicates were performed for each treatment combination. Each replicate comprised different batches of *Ae*. *aegypti* eggs collected from the colony on different days. All trays were checked daily at 09:00, 12:00 and 15:00 hours and water lost through evaporation was replaced. Larvae that had pupated were collected using a plastic pipette, counted and placed in 250 ml plastic cups containing 100 ml deionized water. The sex of pupae was determined by examination of the terminalia using a stereomicroscope. Emergence of adults was recorded daily at the same times used for recording pupation.

### Pupation, emergence, survival and longevity

Time to pupation and time to adult emergence were calculated according to the duration of the development from the first instar until pupal formation and from first instar until the emergence of adults, respectively. Survival to pupation and survival to adult emergence were determined according to the proportion of pupae that survived in relation to the total number of larvae placed in each tray. The production of male pupae was calculated as the number of male pupae collected in the 24 h period after pupation was first observed divided by the total number of male pupae observed in each treatment (total pupation). Sex ratio at 24 h was calculated as male pupae production during the first 24 h in relation to the total number of pupae female in each treatment at 24 h. The overall sex ratio was calculated as the total number of adult males in relation to the total number of adult females that emerged in each treatment.

To determine the longevity of males in each treatment, fifty 24-hour-old males were selected at random, placed in an acrylic cage (30 x 30 x 30 cm) with unlimited access to 10% (wt/vol) sugar solution in a plastic tube with a cotton wick. Another fifty males were placed in an identical cage with a cotton pad treated with pure water. Each treatment was replicated on three occasions. All dead males found each day in each replicate were counted and removed at 09:00 hours.

### Adult body size

Adult males and females from all the treatments were stored at 4°C, and the size of the adults was estimated by measuring their right wing from the distal edge of the alula to the end of the radius vein excluding fringe scales, which is a reliable indicator of body size [20]. The measurement of the wings was carried out to a precision of 0.01 mm using ZEN 2.3 (blue edition) software for the Stemi 508 Stereomicroscope (Carl Zeiss), fitted with a digital camera.

### Adult longevity, fecundity and fertility

For each treatment, 100 females and 100 males were randomly selected and were placed together in acrylic cages of 30 x 30 x 30 cm and supplied *ad libitum* with water and 10% sugar solution. Five days after their introduction into the cage, females were provided with daily access to a blood meal over a period of 14 consecutive days. For this, cattle blood was supplied using a Hemotek PS6B membrane feeding system (Hemotek Ltd., Great Harwood, UK). At 48 hours after the first blood meal, 250 ml plastic containers were placed in each cage with 100 ml of deionized water and a 40 x 4 cm strip of white filter paper as the oviposition substrate. The filter paper strip was removed daily and replaced by another. Eggs were counted and embryonated according to standard procedures (30°C, 48 h) and the fertility was measured by determining the percentage of hatching. Adult mortality was recorded daily by removing and recording the numbers of dead males or females in each cage (treatment), until the last adult had died.

### Flight ability

To determine the flight ability of adults at 24 h post emergence, 100 pupae of one sex were placed in a petri dish (6 cm in diameter and 1.5 cm in height) which was introduced into tubes of 20 cm in height and 7 cm in diameter and placed in an acrylic cage of 30x30x30 cm. Flight ability was measured according to the prevalence of adults that emerged from the pupae and were able to exit the tube into the cage over a 48 h period. This test was performed three times for pupae of both diets and both sexes, regardless of the light rearing treatment.

### Mating capacity

This parameter was estimated as a function of the mating frequency of males. For this, 10 females of the LRD diet were selected at random, and were placed in a cage of 30x30x30 cm. Then, 20 randomly selected males were introduced, 10 from the LRD diet (marked with pink fluorescent powder) and 10 males from the IAEA diet (marked with blue fluorescent powder). The matings were observed directly inside the cages over a period of two hours and the couples were captured to identify the males and record their treatment of origin. These experiments were performed three times. An identical experiment was performed with females from the IAEA diet.

### Statistical analyses

All statistical analyses were performed using the Statistica 7 package (StatSoft Inc., Palo Alto, CA). The effect of diet and light (covered and uncovered trays) on survival to pupation, survival to adult emergence, and male pupae production were analyzed by fitting general linear models (GLMs). Angular transformation (arcsine sqrt) was applied to normalize fertility variables expressed in percentages. GLMs were also fitted to pupation time, emergence time, sex ratio, and flight ability. Means were compared using the Tukey test (P<0.05). To estimate the mean adult male lifespan, the Kaplan-Meier method was used. We also fitted GLMs to analyze the effect of diet and light conditions on fecundity, fertility, longevity and wing length, followed by the Tukey test (P<0.05). The mating frequency of males obtained from larvae that developed on each type of diet was compared by χ^2^ test.

## Results

### Development time

The development time of first instars to pupae differed significantly in males from different diets (F_1,8_ = 35.760; P <0.001) and light conditions (F_1,8_ = 22.790; P = 0.001) (Table 1). Larvae fed with IAEA diet in dark conditions developed significantly faster (mean 3.78 days) than larvae on the LRD diet under light and dark conditions (3.97–4.14 days) (Tukey, P <0.05). No significant differences were observed in first-instar to pupal development times between the female pupae on either diets (F_1,8_ = 1.111; P = 0.323) or light conditions (F_1,8_< 0.001; *P* = 0.995). Time from first instar to adult emergence differed significantly between the diets (F_1,8_ = 26.202; P <0.001) and light conditions (F_3,8_ = 15.645_;_ P = 0.004) in males (Table 1). Male development time was longest in the LRD-light treatment (5.93 ± 0.007 days), shortest in the IAEA diet light and dark treatments (5.54–5.70 days) and intermediate in the LRD-dark treatment. For females, no significant differences were detected between diet treatments in the development time prior to emergence of females (F_1,8_ = 2.441; P = 0.157), although a significant effect of light was observed (F_1,8_ = 12.893; P = 0.007). Female development time to adult emergence was longest in the LRD-light treatment (6.29 ± 0.090 days), shortest in the IAEA-dark treatment (5.98 ± 0.050 days) and intermediate in the LRD-dark and IAEA-light treatments (6.13–6.25 days) (Table 1).

**Table 1:** Mean (± SE) development time in days from first instar to pupae and first instar to adult, on two larval diets: International Agency of Energy Atomic Diet (IAEA) and Laboratory Rodent Diet (LRD).

Treatment	First instar to pupae		First instar to adult	
	Males	Females	Males	Females
IAEA—dark	3.78 ± 0.003^a^	4.42 ± 0.359^a^	5.70 ± 0.009^a^	5.98 ± 0.050^a^
IAEA—light	3.93 ± 0.052^ab^	4.41 ± 0.080^a^	5.54 ± 0.084^a^	6.25 ± 0.064^ab^
LRD—dark	3.97 ± 0.003^b^	4.98 ± 0.003^a^	5.75 ± 0.015^ab^	6.13 ± 0.013^ab^
LRD—light	4.14 ± 0.040^c^	4.70 ± 0.282^a^	5.93 ± 0.007^b^	6.29 ± 0.090^b^

Values followed by different letters (a, b, ab, c) indicate significant differences for comparisons among treatments within each column (ANOVA, Tukey P <0.05).

### Survival of larvae and pupae

The survival of first instar larvae to pupation did not differ significantly between diets (F_1,8_ = 3.225; P = 0.0824) or light treatments (F_1,8_ = 0.625; P = 0.4520). The average proportion of each cohort that survived to pupation varied from 0.94 ± 0.01 to 0.98 ± 0.01 across the different treatment combinations. The same pattern was observed in the survival from first instars to adult emergence, in that neither diet (F_1,8_ = 3.417; P = 0.0731), or light treatment (F_1,8_ = 0.205; P = 0.6631) had any significant effect (range of mean survival 0.93 ± 0.02 to 0.97 ± 0.003).

### Production of male pupae

At 24 h after the beginning of pupation, significant differences were detected in the proportion of male pupae with respect to total production of male pupae in terms of a diet x light treatment interaction (F_1,8_ = 15.556; P = 0.004), although neither of the main effects was significant, indicating a cross-over interaction (Table 2). Insects from the LRD-dark treatment produced a significantly higher proportion of male pupae (93%) during the first 24 h, compared to those observed in the IAEA-dark (83%) or the LRD-light treatment (81%) (Tukey, P <0.05), whereas the IAEA-light treatment was intermediate (Table 2).

**Table 2:** Mean (±SE) proportion of male pupae of *Aedes aegypti* that pupated in the 24 h period after pupation was first observed, pupal sex ratio at 24 h (males/females) and overall adult sex ratio (males/females) fed on IAEA and LRD larval diets.

Treatments	Male pupae production at 24 h	Pupal sex ratio at 24 h	Adult sex ratio
IAEA—dark	0.83 ± 0.02^a^	1.70 ± 0.19^ab^	1.23 ± 0.07ª
IAEA—light	0.86 ± 0.03^ab^	1.76 ± 0.27^ab^	1.17 ± 0.03^ab^
LRD—dark	0.93 ± 0.01^b^	2.33 ± 0.16^a^	1.02 ± 0.04^ab^
LRD—light	0.81 ± 0.01^a^	1.27 ± 0.09^b^	0.98 ± 0.06^b^

Values followed by different letters (a, b, ab) indicate significant differences for comparisons among treatments within each column (ANOVA, Tukey P <0.05).

The 24 h sex ratio differed significantly between light treatments (F_1,8_ = 8.759; P = 0.018) and the interaction of diet x light (F_1,8_ = 6.977; P = 0.030) (Table 2). The highest mean value (2.33 ± 0.16 male pupae per female) was present in the LRD treatment under dark conditions, which was significantly higher than the sex ratio of the same diet (1.27 ± 0.09 male pupae per female) in light conditions (Tukey, P <0.05), but no significant differences were observed between this value and the proportion of male pupae at 24 h for the IAEA diet (1.70 and 1.76 male pupae per female) under either light treatment (Tukey, P >0.05).

The overall adult sex ratio was more male biased in the IAEA diet under dark conditions (1.23±0.07, adult males per female) compared to the LRD diet in light conditions (0.98 ± 0.06) (F_1,8_ = 18.396; P = 0.003), whereas the other two treatments (IAEA-light, LRD-dark) were intermediate (Table 2).

### Adult longevity

The longevity of adult males maintained with water alone did not differ significantly between diet (F_1,8_ = 3.527; P = 0.097) and light treatments (F_1,8_ = 0.202; P = 0.665) and averaged between 4.18 and 4.91 days (Table 3). When adult males had access to sugar solution their longevity increased markedly (mean 37.4–44.8 days) compared to males held with water alone, but did not differ significantly among diets (F_1,8_ = 4.035; P = 0.051), or light treatments (F_1,8_ = 1.708; P = 0.219) (Table 3).

**Table 3:** Mean (± SE) longevity (days) of adults that developed from larvae fed on IAEA and LRD diets. Males in single-sex cages fed with water or sugar solution, and males and females caged together.

Treatments	Males only (water)	Males only (sugar soln.)	Males (caged with females)	Females (caged with males)
IAEA—dark	4.91 ± 0.09^a^	37.43 ± 2.37^a^	29.61 ± 1.07^a^	48.43 ± 2.27^a^
IAEA—light	4.51 ± 0.18^a^	38.18 ± 1.14^a^	27.40 ± 3.55^a^	49.92 ± 2.27^a^
LRD—dark	4.18± 0.41^a^	38.83 ± 1.72^a^	30.61 ± 0.50^a^	53.46 ± 1.38^a^
LRD—light	4.37 ± 0.08^a^	44.77 ± 1.30^a^	28.23± 1.46^a^	48.74 ± 0.49^a^

Values followed by identical letters (a) indicate no significant differences for comparisons of treatments within each column (ANOVA, Tukey P >0.05).

The longevity of adult males that were caged together with females with access to sugar solution did not differ significantly between diet (F_1,8_ = 0.213, P = 0.657), or light treatments (F_1,8_ = 1.333; P = 0.282), with mean longevity values of 27.4–30.6 days, depending on treatment (Table 3). Similar results were observed with respect to adult female longevity when caged with males; no significant differences were observed in the average longevity of females reared on different diets (F_1,8_ = 1.297; P = 0.2877), or light conditions (F_1,8_ = 0.903; P = 0.3672), which ranged between 48.4 and 53.4 days (Table 3).

### Body size (wing length)

**Fig 1:**
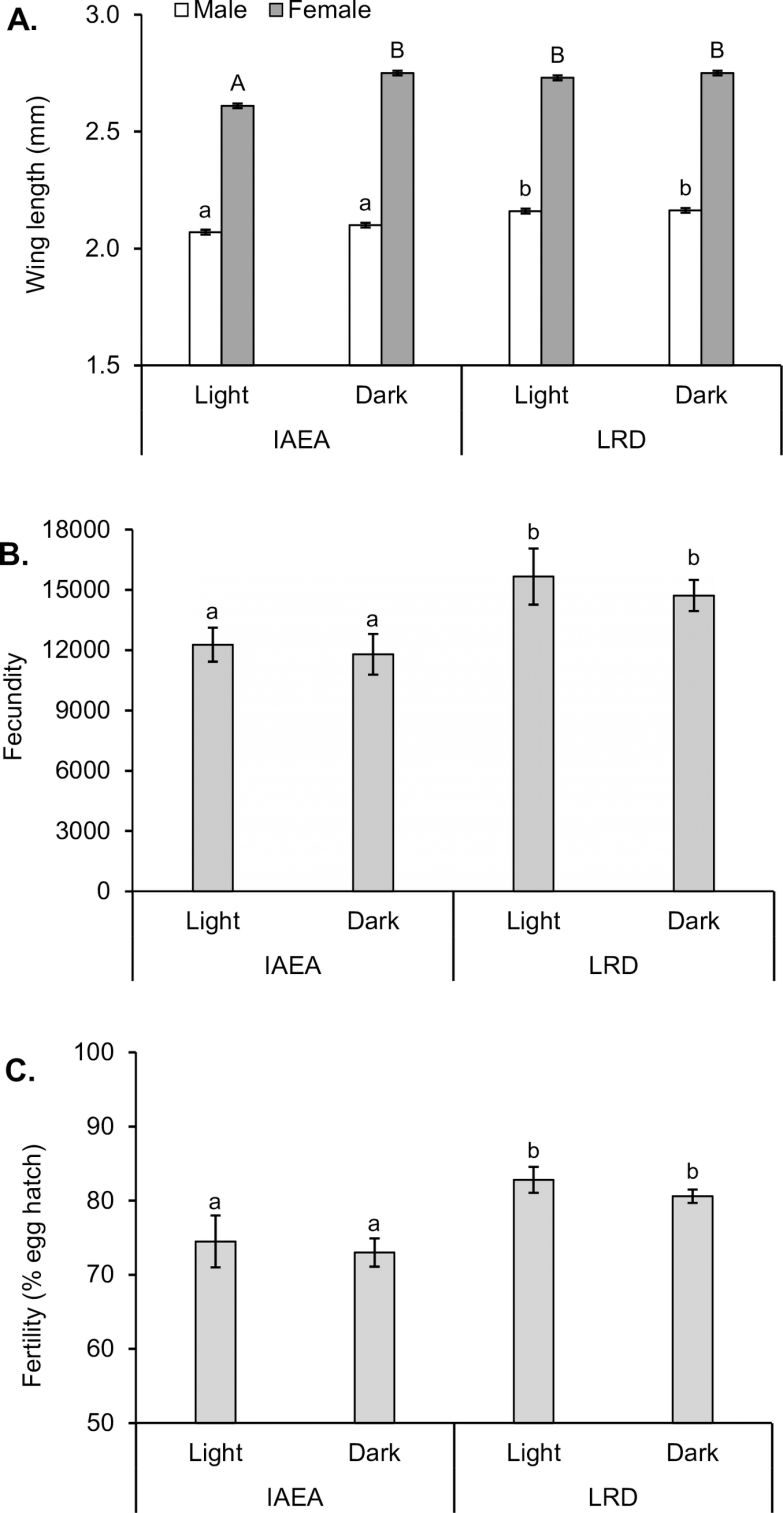
Wing length, fecundity and fertility of *Aedes aegypti* reared during the larval stage on IAEA or LRD diet under light or dark conditions. (A) Mean wing length of adult male (white columns) and female (gray columns) mosquitoes, (B) Mean fecundity expressed as mean number of eggs laid in each cage of 100 female mosquitoes, (C) Mean fertility expressed as percentage of egg hatch. Columns headed by different letters differed significantly for comparisons among treatments for upper case (female) and lower case (male) letters (A), or lower case letters (B, C) (ANOVA, Tukey, P <0.05). Vertical bars indicate SE in all cases.

Mean wing length of males differed significantly with larval diet (F_1,8_ = 66.684; P <0.001), but did not differ with light conditions (F_1,8_ = 3.329; P = 0.106). Males from the LRD-dark and LRD-light treatments (2.16 mm in both cases) had significantly longer wings than those produced with the IAEA-dark and IAEA-lights treatments at 2.10 and 2.07 mm, respectively (Fig 1A).

Female wing length did not differ significantly with diet (F_1,8_ = 4.861; P = 0.059) but did differ significantly with light treatment (F_1,8_ = 9.528; P = 0.015) and their interaction (F_1,8_ = 6.036; P = 0.039). This was because females from the IAEA-light treatment had significantly shorter wings (Tukey, P <0.05) than females from the same diet reared in the dark, or females from the LRD diet reared in light or dark conditions (Fig 1A).

### Fecundity and fertility

The fecundity of females was measured in terms of egg production from each cage of 100 female + 100 male mosquitoes (Fig 1B). The fecundity of females from the LRD diet was ~3,000 eggs higher than females from the IAEA diet (F_1,8_ = 9.300; P = 0.016), whereas the light treatment during the immature stages had no significant effect on adult fecundity (F_1,8_ = 0.468; P = 0.513). The fertility of eggs laid by females, expressed as percentage of egg hatching, was ~80% in the LRD diet treatment, which was ~8% higher than those reared on the IAEA diet (F_1,8_ = 21.488; P <0.001), but light treatment had no significant effect on egg fertility (F_1,8_ = 1.204; P = 0.304) (Fig 1C).

### Flight and mating

The prevalence of flight ability of females reared on the LRD diet (95.3 ± 1.8%) was similar to that of females from the IAEA diet (83.4 ± 4.8%) (F_1,8_ = 1.720; P = 0.226). Similarly, the prevalence of flight ability of males reared on the IAEA diet (92.0 ± 3.5%) was similar to that of males from the LRD diet (89.8 ± 4.1%) (F_1,8_ = 0.179; P = 0.683).

In the mating experiment a total of 60 copulations were observed. The males that were reared on the IAEA diet had a similar number of copulations (N = 31) as males from the LRD diet (N = 29) (*χ*^2^ = 0.820, df = 1, P >0.05).

## Discussion

A series of laboratory studies revealed that *Ae*. *aegypti* reared on a standardized laboratory rodent diet (LRD) during the larval stage, under one of two light regimes, produced similar or slightly fewer male pupae and adults as mosquitoes reared on a diet developed by IAEA researchers. The LRD-reared mosquitoes were generally larger and had a greater reproductive capacity than those reared on the IAEA diet.

Mass production of *Ae*. *aegypti* requires a balanced diet that favors high survivorship, fast and homogeneous larval development, uniformity in body size and production of healthy and high quality males that can compete with wild adult males in a SIT program [21]. The LabDiet 5001^®^ (LRD) is a complete diet formulated for laboratory rodents and is marketed as a standardized diet, with minimal batch variation, for animals undergoing biomedical research. In the Centro Regional de Investigación en Salud Pública LRD has been used for several years as food for the rearing of mosquito larvae *Anopheles albimanus*, *An*. *pseudopunctipennis*, *Ae*. *aegypti* and *Ae*. *albopictus*. The LRD diet had not previously been compared quantitatively with another artificial diet for mosquito larvae. In contrast, the IAEA diet was designed to be used in mass rearing, to provide complete nutrition for the larval development and the production of adult mosquitoes, using inexpensive ingredients that are available globally [12, 22, 23].

The IAEA diet differs from the LRD in that it includes tuna meal, bovine liver powder and brewer’s yeast which are rich in proteins, vitamins, and fatty acids [12, 23]. The ingredients of the LRD diet are rich in protein (fish meal, porcine meat meal, soybean, etc.) carbohydrates (cereals, beet pulp, cane molasses, etc.) and minerals (iron sulfate, manganese oxide, zinc oxide, copper sulfate among others). The bromatological analysis indicated that the LRD had an overall energetic content approximately 10% higher than the IAEA diet (Table 4).

**Table 4:** Bromatological analysis of the of the International Atomic Energy Agency (IAEA) and the Laboratory Rodent Diet (LRD) based on samples of 100 g.

Diet	Humidity	Ashes	Crude protein	Crude fiber	Fat	Carbohydrate	Energetic content	
	(%)	(g)	(g)	(g)	(g)	(g)	Kcalories	Kjoules
IAEA	7.70	15.85	59.22	1.76	5.69	9.77	327.2	1369.0
LRD	11.70	7.34	22.40	3.89	0.96	65.41	359.9	1505.8

Larvae that consumed the IAEA diet had the shortest male pupation time and highest adult emergence between sexes, but no significant diet-based differences were observed in female pupation time. The light treatment exerted a clear influence on male pupation time and emergence of adults of both sexes. Nutritional reserves, particularly glycogen, play a regulatory role in insect development influencing the ability of larvae to pupate. The timing of metamorphosis in *Ae*. *aegypti* larvae is influenced both by the availability of food and by temperature [24]. A short pupation time and pupal development period prior to adult emergence is desirable in a mass rearing program, as these increase the overall rate of insect production. The pupation time and the period prior to emergence of the adults were shorter in *Ae*. *aegypti* compared to that reported for *Ae*. *albopictus* [12], presumably due to species-specific differences in developmental rates.

Following emergence, mosquitoes have teneral reserves of carbohydrates and lipids accumulated in the larval stage, which are used to for adult metabolic requirements [25–27] and flight [28]. In general, female adults had a greater longevity than males. This may be related to the fact that *Ae*. *aegypti* females accumulate greater quantities of carbohydrates and lipids than males [24]. Indeed, the LRD diet contains a higher abundance of carbohydrate-based ingredients but a lower lipid content than the IAEA diet (Table 4).

Of the total production of male pupae in the first 24 hours, 93% were produced from larvae fed with the LRD diet in dark conditions. Similar results in terms of the production of male pupae were reported by Puggioli et al. [12] for the IAEA diet, which led them to consider it cost effective for mass production. In mass rearing it is important to obtain pupae within a brief temporal window to maximize the efficiency of mechanical sex separation procedures applied at the pupal stage [12]. Fast development and emergence of adult males before adult females (protandry) is a form of sexual selection for increased male mating opportunities via access to virgin females [28], so the search for diets that induce the expression of this trait is important.

When males were given access to water their longevity (mean lifespan) was shorter than males that had access to sugar solution. Studies related to the digestion of carbohydrates in mosquitoes have indicated that carbohydrates provide an important source of energy for flight and contribute to longevity and fecundity of mosquitoes [26–28]. The longevity of males that had access to sugar solution was numerically higher in LRD treatments, but no significant differences were observed among the treatments. There was no significant difference between longevity of males caged with other males with free access to sugar, and these values were higher than those of males caged together with females. This decrease in the longevity of males in the presence of females is likely due to the energy demands related to courtship and copula, and possibly increased energy expenditure when living at adult densities that were higher in cages with both sexes (100 mosquitoes/cage) compared to cages with males alone (50 mosquitoes/cage), in addition to usual energetic demands of flight and somatic maintenance. During the larval stages energy reserves are synthesized and accumulated for use in metamorphosis and to provide reserves (lipids and glycogen) for the adult stage. Consequently, the amount of nutrient stored in larvae has important consequences for the longevity of adult males [29, 30]. The values presented in the present work on the longevity of females and males differ markedly with the values for females and males of *Ae*. *albopictus*, even with the same IAEA diet [12], presumably as a result of interspecific variation, or possibly minor methodological variations that had a large impact on adult longevity.

Males reared with the LRD in both light and dark treatments were significantly larger than the males obtained from the IAEA diet in both light and dark treatments. In the case of females, significant differences in size were also observed. The differences in the size of males are likely related to the greater energy content of the LRD diet, since energy intake during the larval stage has a direct influence on adult size in this species [31–33]. Adult size is also positively correlated with dispersal rates of *Ae*. *aegypti* [34], which could contribute to the success of SIT-based vector control.

Significant diet-based differences were detected in fecundity, for which females from the LRD diet produced ~25% more eggs than conspecifics from the IAEA diet (Fig 1B). Fecundity is often related to female body size in *Ae*. *aegypti* [35]. In a previous study on the same IAEA diet [12], lower fecundity was reported for *Ae*. *albopictus* than observed in the present study on *Ae*. *aegypti*. Other comparative studies between *Ae*. *albopictus* and *Ae*. *aegypti*, reported than *Ae*. *aegypti* females lived longer and laid more eggs during their lifetime than *Ae*. *albopictus* females [36].

Dietary carbohydrate is important for egg development [37]. The eggs of the females that developed on the LRD diet had a significantly higher fertility than females from the IAEA diet. Indeed, the LRD diet had a 6-fold higher carbohydrate content and higher energetic content than the IAEA diet (Table 4). Similar patterns in egg fertility were reported for *Ae*. *albopictus* [12].

Despite our initial hypothesis that larval rearing in darkness would increase the time spent feeding and reduce energetic costs of alarm behavior in response to changing light conditions [13–15], the darkness regime had no consistent effects on mosquito traits in the immature or adult stages. Where significant effects were detected these were usually diet-dependent, such as the 24 h production of pupae and pupal sex ratio (Table 2). Given that photoperiod can strongly influence the expression of numerous genes [38], and a broad range of adult behavioral traits [39, 40], there seem to be no clear advantages to mass-rearing rearing *Ae*. *aegypti* larvae in continuous darkness.

Commercial animal diet products have been historically the major components of mosquito rearing diets because they are inexpensive, more homogeneous than natural food sources, and easy to acquire and manage in large quantities [22, 23, 41]. Larval rearing conditions have a direct, and often irreversible effect on adult traits therefore a clearly defined diet is a priority [22]. Considering that the IAEA diet costs approximately US$18.00 per kilogram [12] and the LRD diet costs just US$2.00, and that no significant differences were observed between diets in the survival of pupae and adults, in adult longevity or mating ability in laboratory cages, and since the rate of production of male pupae, fecundity, fertility and adult size was significantly higher in the LRD diet treatment, we suggest that this standardized diet could prove valuable for mass rearing of *Ae*. *aegypti* for use in SIT programs, or other types of vector control programs that require the mass-release of this species.

## Supporting information

S1 File Original data from all experiments. (XLSX)
